# 141. A Blind Spot for Antibiotic Stewardship Programs: Misadministration of Perioperative Antibiotics

**DOI:** 10.1093/ofid/ofab466.343

**Published:** 2021-12-04

**Authors:** Noah Boton, Payal K Patel, Ronald E Kendall, Cheryl Hershey, Mary Jarzebowski

**Affiliations:** 1 University of Michigan, Ann Arbor, Michigan; 2 University of Michigan and VA Ann Arbor Healthcare System, Ann Arbor, MI; 3 VA Ann Arbor Healthcare System, Ann Arbor, Michigan

## Abstract

**Background:**

Hospitalized patients requiring intravenous antibiotics frequently undergo surgical intervention. These surgeries involve multiple transitions of care that may lead to antibiotic delay, additional unnecessary doses, omission, or substitution. While many studies examine the use of antibiotics for surgical site infection prophylaxis, there are no studies investigating antibiotic use in the perioperative period for inpatients already on an IV antibiotic regimen. This study examined the incidence and nature of antibiotic misadministration in the perioperative period among inpatients.

**Methods:**

We conducted a retrospective cross-sectional study at a Veterans Affairs Medical Center involving all inpatients who underwent surgery in 2019. Patients 18 years or older who were on an IV antibacterial regimen prior to surgery were included. Patients undergoing cardiac surgery and patients only receiving surgical infection prophylaxis were excluded. Through manual chart review, we collected information on the prescribed IV antibiotic regimen and timing of antibiotic doses in the perioperative period. Errors were classified as administration of additional unnecessary IV antibiotics and missed, delayed, and additional doses of prescribed IV antibiotics.

**Results:**

There were 168 inpatients on an IV antibiotic regimen who underwent surgery in 2019. Complete data was available for 158 patients. Errors in antibiotic administration in the perioperative period were identified in 64 (41%) patients. Missed, delayed, additional unnecessary antibiotics, and additional doses of prescribed IV antibiotics were identified in 21 patients (13%), 14 patients (9%), 13 patients (8%), and 7 patients (4%), respectively (Figure 1).

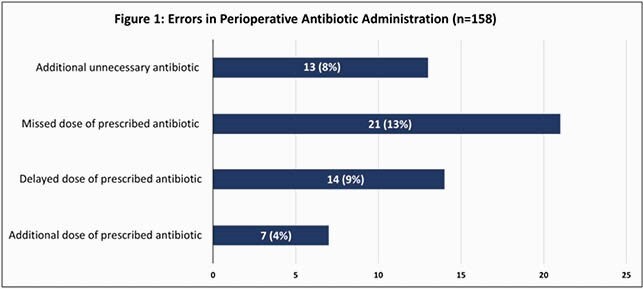

**Conclusion:**

We found errors in antibiotic administration for inpatients undergoing surgery to be common, with the most frequent error being a missed dose of a prescribed IV antibiotic. This illustrates an area for quality improvement in inpatient antibiotic stewardship in our hospital and we suspect in other hospitals as well. Future work will incorporate more centers and examine how these errors affect outcomes for inpatients undergoing surgery, particularly in patients with sepsis or those requiring surgery for infection source control.

**Disclosures:**

**All Authors**: No reported disclosures

